# Benzo[a]pyrene exposure prevents high fat diet-induced obesity in the 4T1 model of mammary carcinoma

**DOI:** 10.3389/fonc.2024.1394039

**Published:** 2024-09-05

**Authors:** Romina Gonzalez-Pons, Jamie J. Bernard

**Affiliations:** ^1^ Department of Pharmacology and Toxicology, Michigan State University, East Lansing, MI, United States; ^2^ Department of Medicine, Michigan State University, East Lansing, MI, United States

**Keywords:** triple-negative breast cancer, obesity, benzo[a]pyrene, high-fat diet, adipose tissue, metastasis

## Abstract

Tumor metastasis is the main cause of death in triple-negative breast cancer (TNBC) patients. TNBC is the most aggressive subtype of breast cancer lacking the expression of estrogen, progesterone, and human epidermal growth factor 2 receptors, thereby rendering it insensitive to targeted therapies. It has been well-established that excess adiposity contributes to the progression of TNBC; however, due to the aggressive nature of this breast cancer subtype, it is imperative to determine how multiple factors can contribute to progression. Therefore, we aimed to investigate if exposure to an environmental carcinogen could impact a pre-existing obesity-promoted cancer. We utilized a spontaneous lung metastatic mouse model where 4T1 breast tumor cells are injected into the mammary gland of BALB/c mice. Feeding a high fat diet (HFD) in this model has been shown to promote tumor growth and metastasis. Herein, we tested the effects of both a HFD and benzo(a)pyrene (B[a]P) exposure. Our results indicate that diet and B[a]P had no tumor promotional interaction. However, unexpectedly, our findings reveal an inhibitory effect of B[a]P on body weight, adipose tissue deposition, and tumor volume at time of sacrifice specifically under HFD conditions.

## 1 Introduction

Obesity is characterized by a state of chronic local and systemic inflammation and exerts significant effects on the development of triple negative breast cancer (TNBC), an aggressive breast cancer subtype that has a high rate of reoccurrence and distant metastasis (1). In obesity, the elevated production of estrogen by adipose tissue affects circulating estrogen levels, and it is widely recognized that these growth-promoting effects of estrogens are essential in the development and progression of human breast cancer. However, estrogen does not have a direct impact on TNBC, since it does not express the estrogen receptor. Metastatic spread of TNBC to distant sites occurs earlier in patients with obesity leading to higher mortality rates (2). The molecular basis for why triple negative tumors are more aggressive in patients with obesity has been explored (3), but the potential impact of excess adiposity on the response to environmental carcinogenic exposure is not well understood. One study demonstrated that obesity promotes carcinogen 7,12-dimethylbenz[*a*]anthracene (DMBA)-initiated mammary tumorigenesis (4). Additionally, epidemiological studies have demonstrated that certain inflammatory conditions can exacerbate carcinogenesis in the context of environmental modifiers. For example, lung cancer is promoted when patients with chronic obstructive pulmonary disease are also cigarette smokers (5). Aflatoxin B exposure synergistically interacts with hepatitis B virus to induce hepatocellular carcinoma (6). Dietary intake of mutagenic compounds in the context of ulcerative colitis promotes colon cancer (7). Herein, we aim to determine if benzo(a)pyrene (B[a]P) exposure promotes lung metastasis in the context of a high-fat diet (HFD), as lung is one of the most common sites of metastatic spread of TNBC (8).

B[a]P is a lipophilic polyaromatic hydrocarbon (PAH) that readily crosses cell membranes and activates the aryl hydrocarbon receptor (AhR) in the cytoplasm. Once activated, AhR is transported into the nucleus where it recognizes and binds to xenobiotic-response elements in gene promoters regulating various PAH-responsive genes. Importantly, AhR target genes include the cytochrome P450 gene family, including *CYP1A1*, and *epoxide hydrolase*, which are able to metabolize B[a]P (9, 10). Once B[a]P is metabolized to BPDE, this metabolite covalently binds to the N2 position of guanines in DNA, forming bulky adducts that can lead to initiating mutations (11, 12). We have previously demonstrated that kynurenine released from adipocytes activates AhR leading to cancer progression *in vitro* (13). How potential endogenous and exogenous AhR ligands may converge to promote cancers has not been explored. We hypothesized that B[a]P enhances the mammary tumor promoting activity of a high fat diet (HFD) leading to tumor progression and metastasis. To test our hypothesis, we subjected the 4T1 transplantation mouse model of TNBC to B[a]P exposure while concurrently feeding a HFD. The host mouse is BALB/c, which expresses the *Ah ^b-2^* allele. The *Ah ^b-1^*, *Ah ^b-2^*, and *Ah ^b-3^* alleles encode AhRs that bind to aromatic ligands like PAHs with high affinity (14).

## 2 Materials and methods

### 2.1 In vivo

Female BALB/c mice were purchased from Charles River Laboratories (MI, USA) at six-weeks of age. Mice were randomly divided into high fat (60% kcal fat); or control diet/low-fat diet (10% kcal fat) (Bio Serve Cat# F3282 and F4031) diet groups (*n=10*). Four weeks after starting the mice on diets, 4T1 cells (4.0 x 10^5^ cells) obtained from American Type Culture Collection (ATCC) were suspended in 50 μL Matrigel (Corning Cat# 354248) and injected into the abdominal mammary fat pad of mice. Five days after 4T1 cells injection, mice received intraperitoneal (IP) injections of either sesame oil (Sigma Cat# S3547) carrier only or benzo[a]pyrene (2 mg/kg/body weight, Sigma Cat# B1760) dissolved in sesame oil for 20 days. 4T1 tumors were measured throughout the study and at sacrifice. Mice were sacrificed 26 days after 4T1 cell injection. Mice were weighed, and mammary and visceral fat pads, tumors, and lungs were excised and weighed. Lungs were removed and inflated with PBS via the trachea and placed in 10% neutral buffered Formalin (Azer Scientific, Cat# PFNBF-20) for 72 hours. Lung specimens in 70% ethanol and frozen mammary adipose tissue were provided to the MSU Histology core for hematoxylin and eosin (H&E) staining. Transverse sections were obtained, and slides were prepared for quantification.

### 2.2 Imaging

Imaging and counting of metastatic sites were performed on an Olympus BX40 microscope. Adipocytes images were analyzed using Image J. Briefly, after importing an image of interest, a known linear scale bar was used to set the scale of the image. The distance of the scale was analyzed to input the distance of the scale bar. The pixel aspect ratio was adjusted to 1, with the unit specified as (µm). Subsequently, background was subtracted, and the images were converted to 8-bit and thresholded to highlight adipocyte areas. Finally, the areas corresponding to adipocytes were measured to quantify adipocytes area. Tissue was randomly sectioned by the histology core and microscopist was blinded to the experimental groups when quantifying lung metastases and adipocyte size.

### 2.3 Kynurenine ELISA

Serum kynurenine was measured by enzyme-linked immunosorbent assay (ELISA), according to manufacturer’s instructions. At sacrifice, blood was collected by cardiac puncture and allowed to clot at room temperature before being centrifuged at 1000 RPM for 5 min. Serum was collected and stored at -20°C. Serum was used for the ELISA to quantify kynurenine levels. Kynurenine ELISA kit was obtained from Immusmol (Bordeaux, France).

### 2.4 Dietary information

Mice were randomly divided into High Fat (60% kcal fat; 20.5% of protein, 36.0% of fat, 3.5% of ash, 0.0% of fiber, <10% of moisture, and 35.7% of carbohydrate) or Control Diet (10% kcal fat; 20.5% of protein, 7.2% of fat, 3.5% of ash, 0.0% of fiber, <10% of moisture, and 61.6% of carbohydrate) groups (Bio Serve Cat# F3282 and F4031) groups. The nutritional composition is similar between diets except for fat and carbohydrate. Tryptophan is obtained from a dietary protein source; since protein composition is 20.5% for both diets, the level of tryptophan in each diet is similar. Food consumption was measured and remained stable across experimental groups.

### 2.5 Statistical analysis

Each bar of tumor size represents the mean ± SEM (*n* = 10). Each bar of body weight represents the mean ± SEM (*n* = 10). The data were evaluated using a two-way ANOVA and were expressed as a mean ± SD. A Tukey’s test was used to compare the means of each treatment/exposure to the means of all other treatment/exposure groups. Values * *p* < 0.05, ** *p* < 0.01, *** *p* < 0.001, **** *p* < 0.0001 were considered statistically significant. Statistical analyses were performed using GraphPad Prism version 10 for Mac, GraphPad Software, La Jolla, CA, USA (www.graphpad.com).

## 3 Results

### 3.1 B[a]P exposure reduces mammary 4T1 tumor volume in HFD-fed mice

**Figure 1:**
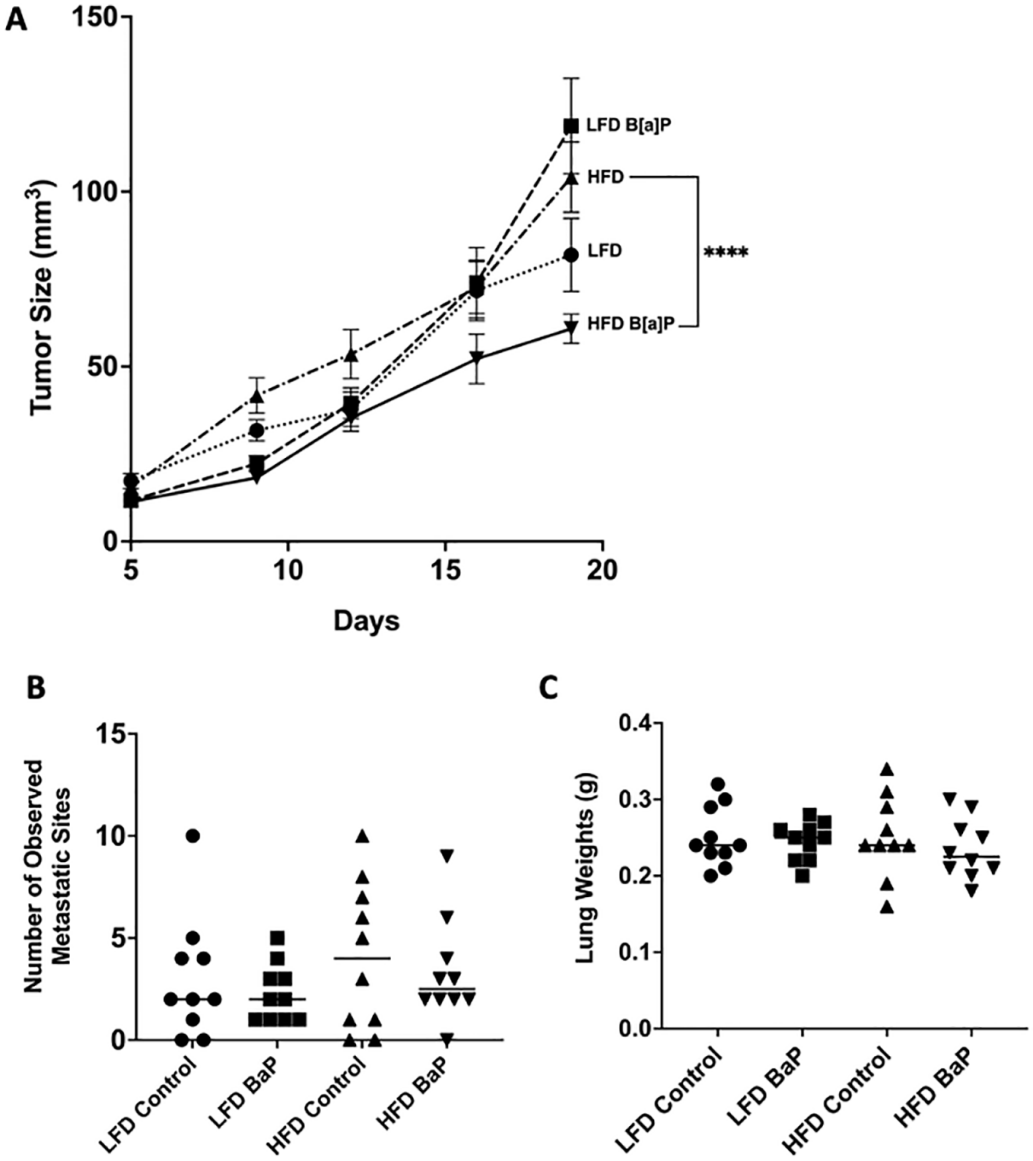
B[a]P-exposed mice on a high-fat diet have significantly reduced tumor volume at time of sacrifice compared to vehicle-exposed mice on a high-fat diet. **(A)** Tumor volume over the last 14 days of diet and B[a]P or vehicle treatment. **(B)** Number of observed metastatic sites quantitated under the microscope. **(C)** Lung weights measurements at sacrifice. Each bar represents the mean ± SEM (n = 10 mice/group). A two-way ANOVA was performed to determine statistical significance and the value ****p < 0.0001 is considered statistically significant.

BALB/c 4T1 tumor bearing mice were fed respective diets and exposed to either B[a]P or vehicle treatment for 26 days. The volume of tumor at sacrifice was not significantly larger in LFD-fed compared to HFD-fed mice or LFD-vehicle compared to LFD-B[a]P. However, opposite to what was hypothesized, B[a]P exposure significantly reduced the effect of HFD on tumor volume at time of sacrifice (p<0.0001). The number of lung metastases were not affected by diet and/or B[a]P exposure (**Figure 1B**). Statistical differences in lung weights by diet and/or B[a]P exposure were not detected (**Figure 1C**). The weights of the kidney, spleen, and liver were not significantly impacted by diet and/or B[a]P exposure, even when normalized to bodyweight (data not shown).

### 3.2 B[a]P reduces body weight in HFD-fed mice

**Figure 2:**
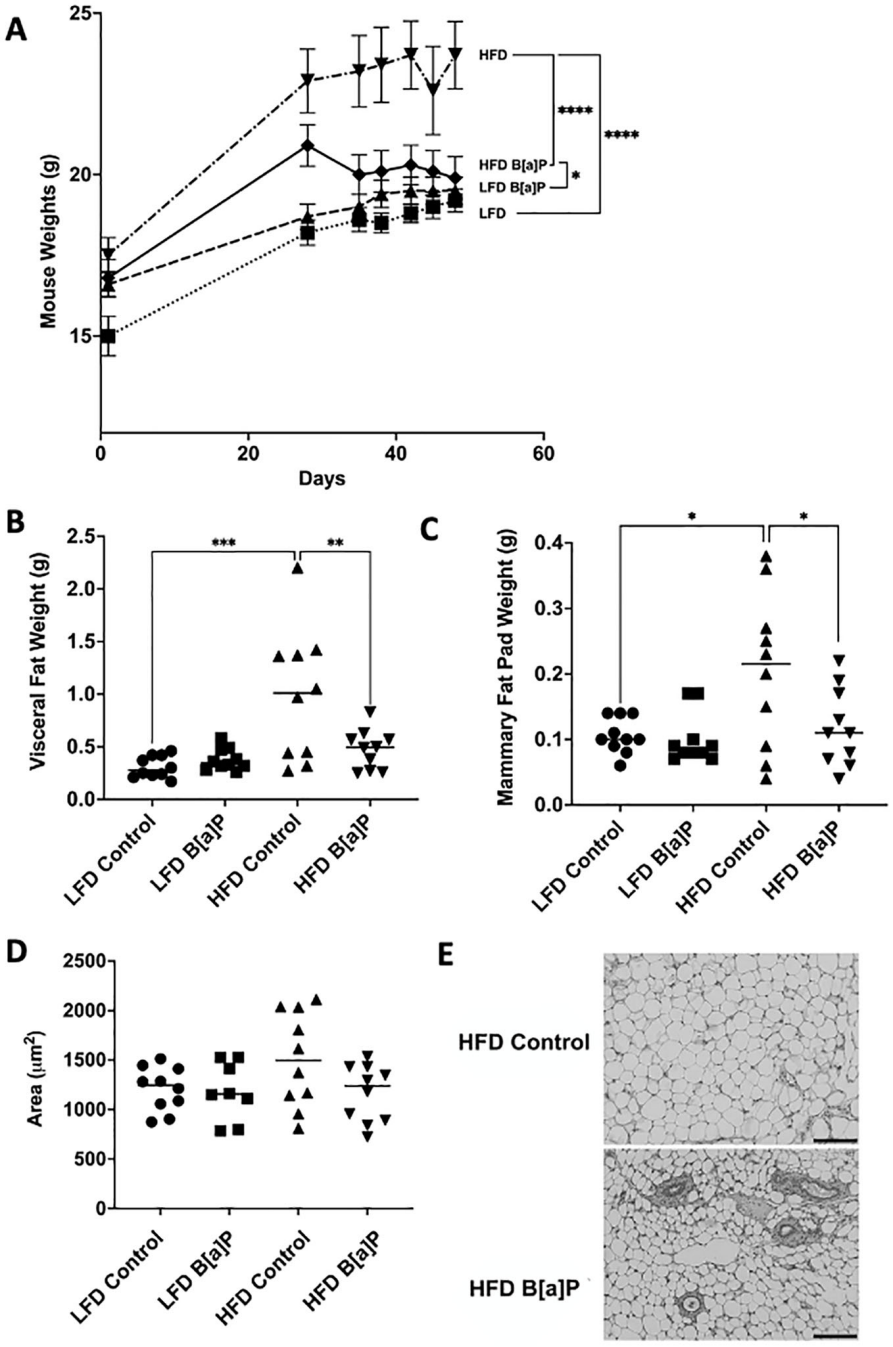
B[a]P exposed mice on a high-fat diet have significantly reduced body weight, visceral fat pad weight, and mammary fat pad weight at time of sacrifice compared to vehicle exposed mice on a high-fat diet. **(A)** Mouse body weights over the course of the study. Mice were on respective diets and were administered B[a]P or vehicle. **(B, C)** Visceral and mammary fat pad weights at sacrifice, respectively. **(D)** Analysis of mammary adipocytes size in hematoxylin and eosin in stained mammary fat pad. **(E)** Microscopy of mammary adipose tissue; scale bar 140µm. Each bar of body weight represents the mean ± SEM (n = 10 mice/group). A two-way ANOVA was performed to determine statistical significance and values *p < 0.05, **p < 0.01, ***p < 0.001, ****p < 0.0001 are considered statistically significant.

The body weights of mice fed a HFD were significantly greater compared to those of mice fed the LFD at the time of sacrifice (p<0.0001) (**Figure 2A**). The body weights of B[a]P-exposed mice fed a HFD were significantly greater compared to B[a]P-exposed mice fed a LFD at the time of sacrifice (p<0.05) (**Figure 2A**). Interestingly, the body weights of B[a]P-exposed mice fed a HFD were significantly lower compared to vehicle-exposed mice fed a HFD at the time of sacrifice (p<0.0001) (**Figure 2A**). HFD-fed mice had a significantly increased visceral adipose tissue (**Figure 2B**) and mammary adipose tissue (**Figure 2C**) weight compared to mice on the LFD (p<0.001). Consistent with the reduced total body weight, HFD-fed mice exposed to B[a]P had a significantly reduced visceral (**Figure 2B**) and mammary (**Figure 2C**) adipose tissue weight than HFD-fed, vehicle exposed mice (p<0.01). A reduction in average mammary adipocyte size was observed in HFD-fed mice exposed to B[a]P compared to HFD-fed vehicle exposed mice, although not significant (**Figure 2D**). Images show mammary adipose tissue (**Figure 2E**).

### 3.3 Serum kynurenine concentration is significantly increased in HFD-fed mice exposed to B[a]P

**Figure 3:**
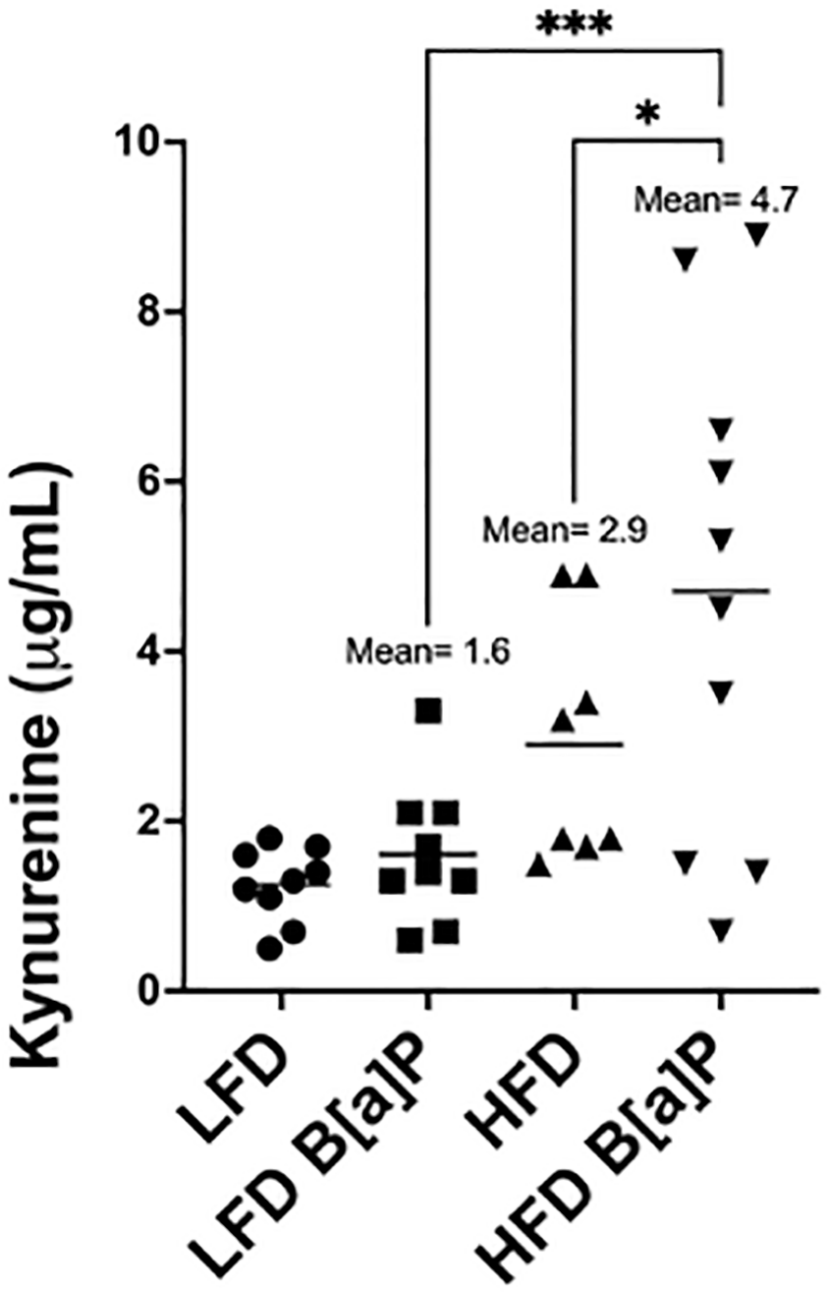
Serum kynurenine concentration is significantly increased in HFD-fed mice exposed to B[a]P. Serum kynurenine concentrations as measured by ELISA. A two-way ANOVA was performed to determine statistical significance and values *p < 0.05 and ***p < 0.001 are considered statistically significant.

Serum kynurenine levels were not significantly different in HFD-fed mice when compared to LFD-fed mice (**Figure 3**). Serum kynurenine levels were not significantly different between B[a]P or vehicle exposed mice on LFD (**Figure 3**). Only the combinatorial effect of B[a]P exposed mice on a HFD significantly increased serum kynurenine compared to all other groups. B[a]P exposed mice fed a HFD had significantly higher levels of serum kynurenine compared to B[a]P exposed mice on a LFD (p<0.001) or vehicle exposed mice on a HFD (p<0.05) (**Figure 3**).

## 4 Discussion

High-fat diets are a major contributor to obesity and a strong risk factor for triple-negative breast cancer (TNBC) (15–19). Herein, the HFD-fed mice developed tumors that were not significantly different in volume compared to those of LFD-fed mice or LFD-vehicle compared to LFD-BaP suggesting no tumor promotional effect of diet or B[a]P, individually in this study. (**Figure 1A**). This result may be due to the shorter duration of HFD-feeding compared to previously published study which demonstrated that long-term (16 weeks) intake of a high-fat diet increases tumor growth of TNBC cells (4T1 cells) in BALB/c mice (20). Our rationale for only feeding the HFD for 4 weeks prior to B[a]P exposure was based on our hypothesis that there would be an interaction between diet and B[a]P exposure on tumor promotion and metastasis. Herein, we show that B[a]P exposure was associated with reduced body weight, reduced overall mammary and visceral fat weight and reduced tumor volume only when the 4T1 tumor-bearing mice were challenged with a HFD, suggesting that B[a]P inhibited HFD-promoted weight gain. Due to the lipophilicity of polyaromatic hydrocarbons such as B[a]P, bioaccumulation in adipose tissue has been observed in several species (21) and in mammary glands (22). However, the adipose tissue reducing mechanism of B[a]P observed herein is unknown.

B[a]P has a wide range of biological activities. B[a]P interacts with the AhR, induces reactive oxygen species (ROS), forms DNA adducts, promotes immunosuppression, modulates the microbiome, and induces epigenetic changes. Many of these mechanisms have been shown to influence adiposity and/or inflammation. B[a]P can promote hematotoxcity which may induce immunosuppression and impair HFD-promoted inflammatory responses and adiposity (23). Another AhR-independent mechanism may involve the translocation of hormone-sensitive lipase, a critical component of the lipolytic pathway responsible for fat breakdown. This translocation is stimulated by an increase in ROS in adipose tissue, highlighting the essential role of ROS in the complete process of lipolysis (24, 25). Therefore, further exploration of the translocation of hormone-sensitive lipase and its stimulation by ROS in visceral and mammary adipose tissue could contribute to a more comprehensive understanding of the adipose tissue-reducing effects of B[a]P. In addition to impacting adipogenesis, B[a]P could directly influence cancer cells by unknown mechanisms. Other benzo-derivatives have apoptosis-inducing activities in cancer cells. Anti-tumor effects have been identified for benzopyran derivatives (26–28) and a novel benzocoumarin-stilbene hybrid (29).

The gut microbiome has emerged as a key player in adiposity regulation. Certain microbial species have been associated with obesity, while others have been linked to leanness. The microbiome can influence adiposity through various mechanisms, including the production of metabolites that affect energy balance and inflammation. For example, the short-chain fatty acid butyrate has been shown to promote energy expenditure and reduce adiposity (30, 31). Primary findings show that butyl butyrate was significantly altered in human microbiota by B[a]P exposure (32) suggesting that B[a]P leads to changes in the abundance of volatile metabolites in the microbiota which can impact adiposity. Future studies could aim to identify microbial species impacted by B[a]P exposure, elucidating their specific contributions to energy metabolism, inflammatory responses, and adiposity regulation. For instance, experiments could involve microbial community profiling using high-throughput sequencing techniques to assess changes in species diversity and abundance in response to B[a]P exposure. All these potential mechanisms potentially outweigh that of AhR-dependent effects.

Interestingly, B[a]P-exposed mice on a HFD were protected from obesity closely resembling the phenotype observed in studies where mice are deficient in AhR activity. For example, AhR knockout mice are protected from HFD-induced obesity (33). This effect was only observed when challenged with HFD—no effect on weight gain was observed in LFD-fed mice. Adipose tissue-specific depletion of AhR, protected against diet-induced obesity (34). Consistent conclusions are drawn from alternative models: chemical inhibition of AhR by either α-napthoflavone or CH-223191 protected against Western diet-induced weight gain *in vivo* (35). Therefore, there may be a mechanism whereby B[a]P has AhR inhibitory activity by either *1)* reducing the activity of endogenous agonists from adipose tissue, such as kynurenine, and/or *2)* inducing the expression of the AhR repressor.

Several conclusions can be drawn from this study to be applied to future experiment design. Although our prior studies demonstrated that HFD-feeding of mice significantly increased adipose tissue kynurenine (36), herein, circulating kynurenine concentration was not associated with overall adipose tissue weight. The highest concentrations of serum kynurenine were observed in HFD-fed, B[a]P exposed mice (**Figure 3**)—a group that had significantly less body weight and adipose tissue weight compared to the HFD-fed, vehicle exposed mice. The elevated concentration of serum kynurenine in B[a]P exposed mice on a HFD may be more reflective of the amount of total body AhR activation and/or tumor AhR activation, as the transcriptional activity of AhR induces indoleamine 2,3-dioxigenase (IDO), the enzyme responsible for the catabolizing tryptophan to kynurenine. Elevated IDO leads to increases in kynurenine in a positive feedback manner (37). Tumor cells exhibit a high demand for nutrients, particularly tryptophan, to promote their growth and are capable of releasing kynurenine into the periphery (37). Therefore, elevated levels of serum kynurenine in the B[a]P-exposed group fed a HFD may be linked to the secretion of kynurenine from tumors. To further explore these findings, future studies could test this hypothesis in different mouse strains that have higher and lower affinity AhR alleles to see if we observe the same modulatory effect with respect to kynurenine levels and/or adipose tissue mass in B[a]P-exposed mice challenged with HFD.

We had originally hypothesized that there would be a positive interaction between B[a]P and HFD on progression and metastasis. One study limitation is that 4T1 model of mammary carcinoma is highly aggressive, potentially missing a more sensitive window for detecting interactions. Our hypothesis may be better suited to be tested in transgenic models where tumor formation occurs over the course of months instead of weeks. However, by utilizing the 4T1 model of tumorigenesis, we determined there was no additional effect of B[a]P on the promotion of established cancer. Instead, we unexpectedly discovered that B[a]P had an off-target effect, inhibiting HFD-promoted adipogenesis.

## Data Availability

The raw data supporting the conclusions of this article will be made available by the authors, without undue reservation.
